# Do Most Children with Functional Constipation Meet the Commonly Used Clinical Trial Endpoints?

**DOI:** 10.3390/children12020234

**Published:** 2025-02-15

**Authors:** Samantha Arrizabalo, Carlos Alberto Velasco-Benitez, Daniela Alejandra Velasco-Suarez, Rafael Giner, Miguel Saps

**Affiliations:** 1 Department of Pediatrics Gastroenterology, Hepatology, University of Miami, Miami, FL 33136, USA; 2 Department of Pediatrics, Universidad del Valle, Cali 76001, Colombia

**Keywords:** children, adolescents, functional constipation, Rome criteria

## Abstract

Background/Objectives: Functional constipation (FC) is diagnosed using the Rome IV criteria, which require at least two of seven symptoms for diagnosis. Clinical trials evaluating FC treatments commonly use bowel movement frequency, stool consistency, and fecal incontinence as primary endpoints. However, there is limited data on whether these endpoints accurately represent the symptom distribution in children with FC. This study assessed the frequency of each criterion in a large children’s community sample to determine whether commonly used clinical trial endpoints accurately reflect symptom distribution. Methods: A cross-sectional study of school children aged 8–18 years was conducted across seven Colombian cities. Participants completed the Pediatric Gastrointestinal Symptoms Rome IV Questionnaire (QPGS-IV). The prevalence of FC and the distribution of diagnostic criteria were analyzed, calculating the percentage of each criterion. Results: 6611 children completed the questionnaires. FC was diagnosed in 12.8% of participants, making it the most common disorder of gut–brain interaction. The most reported criteria were fewer than two stools per week (66.1%) and painful bowel movements (65%), while fecal incontinence was uncommon (6.9%). 60.5% of participants met only two criteria, with two or fewer defecations per week and painful bowel movements being the most common combination. Conclusions: This study reveals significant variability in Rome IV criteria prevalence for FC, highlighting disparities between the most common endpoints in clinical trials and symptom distribution in a community-based cohort. Painful bowel movements emerged as a critical diagnostic component but remain underutilized as an endpoint in pediatric trials. These findings suggest the possible need to reassess endpoint selection in clinical trials.

## 1. Introduction

Functional constipation (FC) is one of the most common disorders of gut–brain interaction (DGBI) in children, with an estimated overall prevalence of 9.5% [1,2]. It falls under the category of functional defecation disorders, which also includes infant dyschezia and functional non-retentive fecal incontinence [3]. Beyond its high prevalence, FC significantly impacts children’s quality of life (QoL) by causing physical discomfort, social withdrawal, and emotional distress [4]. The persistent nature of symptoms often leads to reduced school attendance and limitations in daily activities, further affecting overall well-being. As such, FC frequently prompts general pediatric visits and referrals to pediatric gastroenterologists [5].

FC is diagnosed using the pediatric symptom-based Rome IV criteria. These criteria have been updated in 2016 and used for both research and clinical purposes [5]. To meet the criteria for FC, children have to meet at least two out of six criteria once a week for one month, with one of them being composed criteria: painful or hard stool [6]. Thus, there are a total of seven items in the criteria (Table 1) [7].

In clinical trials evaluating FC treatments, endpoints often include a single or a combination of items from the Rome criteria [8,9,10,11]. Selecting appropriate endpoints should consider both clinical relevance and their frequency among children with FC to ensure adequate representation. Advancing the knowledge of the frequency of each criterion has important implications for research and daily practice. Differences in the frequency of each criterion at the time of inclusion into a clinical trial can impact the ability to recruit or to meet success criteria (i.e., a child with a normal frequency of bowel movements or normal consistency can potentially be included in a clinical trial that uses the Rome diagnosis as inclusion criteria).

Among the Rome IV criteria, some of them have been more frequently selected as endpoints than others. For instance, the primary endpoint in the pediatric clinical trial on linaclotide (successfully met endpoints) [9] was the change in frequency of bowel movements from baseline (in line with FDA recommendations in adults), while the pediatric trial on prucalopride (failed to meet endpoints) used a combined endpoint of frequency of bowel movements and episodes of fecal incontinence [10,11]. It remains unclear whether the selection of endpoints for these pediatric trials could explain the difference in outcomes with studies conducted in adults [12,13,14].

These variations also have practical implications. Not all children with FC exhibit the same diagnostic criteria. Fecal incontinence, a criterion with a high impact on children and families, is not present in all children who meet the diagnostic criteria for FC. Moreover, not every child that meets the Rome criteria for FC has a low frequency of bowel movements. Similarly, stool consistency, which is frequently used as a secondary endpoint, has been preferred in pediatric trials despite its variability [9,15,16].

To standardize FC management, scientific societies such as the European Society for Paediatric Gastroenterology Hepatology and Nutrition (ESPGHAN) and the North American Society for Paediatric Gastroenterology Hepatology and Nutrition (NASPGHAN) have published guidelines for the treatment of FC in children [17]. They aim to provide evidence-based recommendations to standardize the diagnosis and management of FC, ensuring consistency in clinical practice and improving patient outcomes. These guidelines emphasize adequate fiber and fluid intake, physical activity, and pharmacologic treatments (oral osmotic and stimulant laxatives, stool softeners, and enemas) [18]. However, there is a paucity of evidence for some of these interventions [19], and treatment outcomes have often been suboptimal, as seen in large clinical trials with negative results. Lubiprostone, a chloride channel activator, increases intestinal fluid secretion to facilitate stool passage, while prucalopride, a serotonin (5-HT4) receptor agonist, enhances colonic motility. However, neither has demonstrated significant benefits for FC in children [8,10]. These shortcomings underscore the need for clinical trials for the various interventions commonly used and the publication of guidelines for clinical trials for FC in children using adequate endpoints.

Data on the frequency of each individual criterion, particularly those that have been most commonly selected as endpoints in pediatric FC, is limited. This study addresses this gap by analyzing the frequency of each criterion among a large sample of community children with FC.

We hypothesized that most children and adolescents diagnosed with FC meet at least one of the most commonly used endpoints (frequency, consistency, and fecal incontinence), and only a minority of children will meet all three criteria.

## 2. Materials and Methods

### 2.1. Participants Recruitment

Children and adolescents were recruited from eight public and two private schools in seven cities dispersed throughout Colombia (Bucaramanga, Corozal, Tulua, Florencia, Palmira, La Union, and Cali).

Inclusion criteria: students aged between 8 and 18 years of age. Exclusion criteria: children with a history of organic diseases.

### 2.2. Consent and Assent

Before participating, caregivers gave consent, and children provided assent. Consent forms were sent to caregivers through the students. Child assent was obtained after parental consent was secured, and only those with both signed forms were enrolled in the study.

### 2.3. Data Collection

Participants completed the self-report form of a validated questionnaire to diagnose DGBI in children, the Questionnaire Pediatric Gastrointestinal Symptoms (QPGS-Rome IV) [20]. Parents were not permitted to complete the questionnaire. Prior to completing the questionnaires, a member of the research team provided instructions on their completion and was then available to clarify questions.

### 2.4. Statistical Analysis

The study was approved by the Ethics Committee of the Hospital Universitario del Valle “Evaristo García” in Cali, Colombia (024-2019, 27 February 2020).

### 2.5. Ethical Approval

Data of children meeting the diagnosis for FC was selected for analysis. Demographic data were analyzed using measures of central tendency, including mean and standard deviation (SD). For categorical variables, frequency and percentage were calculated for each criterion. To compare the groups, 2 × 2 contingency tables were used, with Fisher’s exact test applied when appropriate. Statistical significance was set at <0.05.

## 3. Results

A total of 6797 school-aged children and adolescents participated in the study, 186 were excluded due to organic diagnoses or lack/incomplete survey completion. Data on 6611 (97.2%) children were analyzed. The analysis of the sample showed that 23.4% (1552) of school-aged children and adolescents were diagnosed with at least one DGBI. Among them, 68.2% (1059) were female (*p* = 0.00), 72.1% (1119) were adolescents (*p* = 0.01,) and 51.4% (798) were from Cali (*p* = 0.00) (Supplementary Table S1).

**Figure 1:**
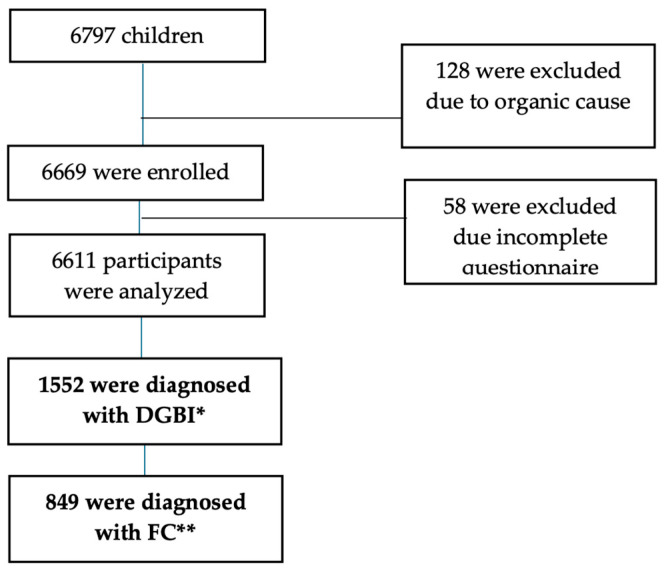
Flowchart of study enrollment for children and adolescents. * Disorders of gut–brain interaction (DGBI); ** functional constipation (FC).

FC was the most common DGBI (849, 12.8%) (Figure 1). The majority of participants were adolescents (70.1%) with a mean age of 13.7 ± 2.2 years; 81.4% attended public schools, 66.1% were females and 51.4% were identified as mixed race (Table 2 summarized the demographics characteristics)

**Figure 2:**
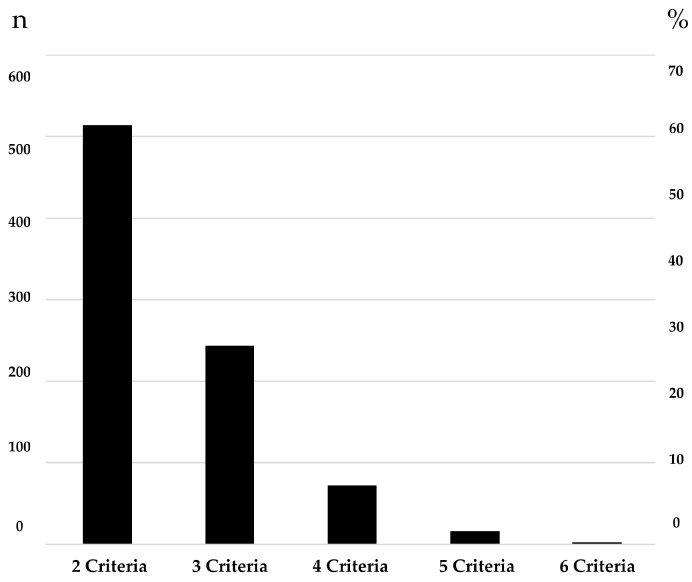
Distribution of participants by number of criteria met (n = 849).

**Figure 3:**
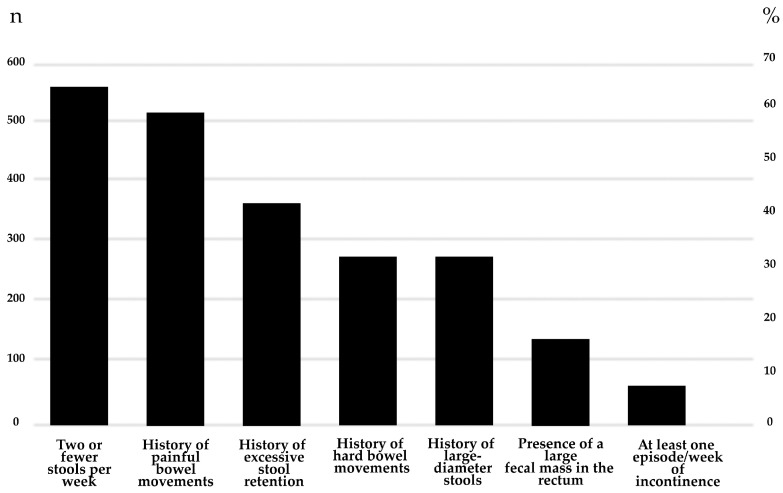
Prevalence of individual Rome IV diagnostic criteria for FC (n = 849).

Most participants diagnosed with FC met two diagnostic criteria (514, 60.5%); 244 (28.7%) met three criteria, and only 3 participants (0.4%) met six criteria (Figure 2 summarizes the distribution of participants by number of criteria met). None reported having the seven items of the diagnostic criteria. The most commonly reported criterion was having two or fewer stools per week, reported by 66.1% of participants. The least frequently reported criterion was fecal incontinence of at least “once a week” (59 children, 6.9%) (Figure 3 presents the prevalence of individual Rome IV diagnostic criteria for FC (n = 849). We found statistically significant differences in age but not in gender among participants reporting fecal incontinence: 31 (52.5%) were adolescents and 28 (47.5%) were school-aged children (*p* = 0.00), while 35 (59.3%) were female, and 24 (40.7%) were male (*p* = 0.25).

The most common combination of criteria was the presence of less than two stools per week and a history of painful bowel movements that was found in 127 out of the 849 children (15%) with FC. The combination of low frequency and hard consistency criteria was uncommon; 57 (6.7%) participants had fewer than two stools per week and a history of hard bowel movements. Five (0.6%) children reported fewer than two bowel movements per week along with fecal incontinence occurring at least once per week. Only one child had hard bowel movements and fecal incontinence at least once weekly. None of the children or adolescents met all three criteria for frequency, consistency of bowel movements, and fecal incontinence.

## 4. Discussion

Our study found that DBGI and FC in particular were common in children and adolescents. Approximately one in eight school children met the criteria for the diagnosis of FC. These findings align with previous epidemiological studies reporting the prevalence of FC ranging from 9% to 17% in pediatric populations [21,22]. Additionally, a systematic review by Bloem et al. (2025) estimated a prevalence of 11.39% (95% CI: 9.34–14.11%) among children aged 4–18 years [3]. While socioeconomic and regional factors are known to influence the prevalence of FC [23], there is limited data on how symptoms are reported and perceived across different populations. Understanding these variations is important for refining clinical guidelines to ensure accurate diagnosis and effective treatment strategies.

Despite the high prevalence of FC, there is a paucity of treatments approved by the regulatory agencies. Thus, there is a great need for well-designed clinicals. A deeper understanding of the frequency of each individual criterion of the Rome criteria is of particular importance at the time of selecting inclusion criteria and clinical endpoints for clinical trials.

Most children in the study had at least one of the three most widely selected endpoints in placebo-controlled trials (frequency of bowel movements, consistency of stools, and episodes of fecal incontinence) [9,11,24]. No children met the three endpoints. As the diagnosis of FC requires meeting at least two criteria, we were interested in investigating what percentage of children with FC met only two criteria. We found that 60.5% of children with FC were diagnosed based exclusively on meeting two criteria, with the most common combination being the low frequency of bowel movements and painful stools, reported by 127 participants in our sample. This suggests that among children in a clinical trial, the improvement of a single criterion will likely result in no longer meeting the Rome criteria for FC. This was the success criteria recommended by the subcommittee of clinical trials for FC of Rome IV [25].

Frequency was the most common criterion in children with FC. This was the primary endpoint of the study on linaclotide that resulted in its approval by the FDA for the treatment of FC in children (the only drug approved in the US so far) [9]. In contrast, the prucalopride trial required meeting two endpoints for success (responder definition included changes in the frequency of bowel movements and episodes of fecal incontinence), and the trial did not show superiority compared to placebo in children but did show efficacy in adults [12,13,26]. By the end of the trial, children in the prucalopride trial had approximately twice as much frequency of fecal incontinence as those in our sample. Children in the negative lubiprostone placebo-controlled study also had a higher frequency of fecal incontinence than our sample [8]. Comparing the data of the prucalopride and the lubiprostone studies with our community data may help provide some insight into the severity of symptoms among children enrolled in those clinical trials and its possible relation to the lack of success. The fact that only 59 out of 849 children (6.9%) in our sample had fecal incontinence could suggest that children in the prucalopride [10] and lubiprostone [8] trials could have had more “severe” constipation, which may help explain the lack of treatment success in those studies. It is unclear if the difference reflects greater severity of symptoms in children volunteering to be part of a study compared with community children, whether it is the result of regional variations or just the result of chance.

Painful bowel movements were the second most reported criterion. We found that painful bowel movements were almost as common as low-frequency bowel movements and twice more frequently reported than hard stools. Interestingly, none of the large clinical trials have investigated changes in painful stools while frequency and consistency have been the primary or secondary endpoints in all clinical trials [9,15,16,24]. Since pain might significantly affect children’s well-being, assessing its impact on QoL could provide valuable insight into treatment success. Given its role in treatment-seeking behavior, practitioners should evaluate and address pain-related distress in pediatric FC. Incorporating pain severity as a key factor in treatment response may enhance future guidelines, ensuring a more comprehensive approach to pediatric FC management. Additionally, further research should investigate whether assessing painful stools could serve as a meaningful clinical trial endpoint to assess patient-oriented meaningful treatment success.

The pathophysiology of FC in children is incompletely understood. It is thought that children frequently retain stools that, in turn, become dry and hard, leading some children to overflow incontinence. The fact that only a few children had a combination of stool retention, hard stools, and fecal incontinence either suggests a poor understanding of the questions of the QPGS-IV or that alternative mechanisms could explain the signs and symptoms of a large proportion of children with FC.

Some of the strengths of our study include the very large sample of community children and the wide geographical, ethnic/racial, and socioeconomic representation of the sample. Socioeconomic, cultural, and regional variability may influence the reporting of symptoms. The data of this study may help design regional treatment strategies. Still, our study is not devoid of limitations, including the inability to assure the external validity of our sample. The fact that children did not undergo physical examinations is an additional limitation of our study. However, the latter is a relative limitation as the QPGS is a standalone questionnaire recommended by the Rome IV criteria to diagnose DGBI without the need for a physical exam. Additionally, selection bias may have influenced our findings, as our community-based sample may not fully represent children with more severe or treatment-resistant FC, such as those who are possibly more likely to be enrolled in clinical trials. Furthermore, recall bias could be a factor, as self-reported data from the QPGS-IV questionnaire may introduce inaccuracies in symptom reporting.

## 5. Conclusions

FC is a common disorder in community children. Low frequency of bowel movements and painful stools are the most common criteria reported by children with FC. The impact of painful stools on quality of life remains underexplored and should be further investigated. Given its clinical significance, pain could be considered as a potential endpoint in future clinical trials.

Future research should determine whether current trial endpoints adequately reflect the clinical burden of FC in children. Our findings provide valuable insights into symptom prevalence and endpoint selection, which may inform future clinical trials. Further validation across diverse populations is needed to assess the reliability of our findings.

## Figures and Tables

**Table 1:** Pediatric Rome IV criteria for functional constipation [6].

To meet the diagnostic criteria, >2 symptoms must be present for >1 month prior to diagnosis: Two or fewer defecations per week >1 episode of fecal incontinence per week History of retentive posturing or excessive volitional stool retention History of painful or hard bowel movements Presence of a large fecal mass in the rectum History of large-diameter stools that may obstruct the toilet

**Table 2:** Demographics characteristics (n = 849).

Demographics	n (%)
**Age (years)**	
X ± SD	13.7 ± 2.2
Range	8–18
**Sex**	
Female	561 (66.1)
Male	288 (33.9)
**Age groups**	
School age (8–12 years)	254 (29.9)
Adolescents (13–18 years)	595 (70.1)
**Race**	
Mixed	366 (51.4)
White	217 (30.5)
Black	96 (13.5)
Indigenous	33 (4.6)
**School**	
Public	691 (81.4)
Private	158 (18.6)
**City**	
Bucaramanga	21 (2.5)
Corozal	65 (7.7)
Cali	424 (49.9)
Palmira	87 (10.3)
Tulua	201 (23.7)
Florencia	27 (3.2)
La Union	24 (2.8)

## Data Availability

The data presented in this study are available on request from the corresponding author. The data are not publicly available due to privacy and ethical restrictions.

## Supplementary Materials

The following supporting information can be downloaded at: https://www.mdpi.com/article/10.3390/children12020234/s1, Table S1: Cohort Comparison.

## Abbreviations

The following abbreviations are used in this manuscript:

FC	Functional constipation
DGBI	Disorder of gut–brain interaction
